# Investigation of Trajectory Tracking Control in Hip Joints of Lower-Limb Exoskeletons Using SSA-Fuzzy PID Optimization

**DOI:** 10.3390/s25051335

**Published:** 2025-02-22

**Authors:** Wei Li, Xiaojie Wei, Dawen Sun, Siyu Zong, Zhengwei Yue

**Affiliations:** 1 College of Mechanical and Vehicle Engineering, Changchun University, Changchun 130022, China; 18763331478@163.com (X.W.); 15981067409@163.com (S.Z.); 2 Key Laboratory of Intelligent Rehabilitation and Barrier-Free Education for Disabled Persons, Ministry of Education, Changchun 130022, China; 3 College of Computer Science and Technology, Changchun University, Changchun 130022, China; 13904455216@163.com; 4 Shandong Jite Industrial Technology Co., Ltd., Rizhao 276800, China; 13450271031@163.com

**Keywords:** lower-limb exoskeleton, Sparrow Search Algorithm, fuzzy PID control, trajectory tracking

## Abstract

The application of lower-limb exoskeleton robots in rehabilitation is becoming more prevalent, where the precision of control and the speed of response are essential for effective movement tracking. This study tackles the challenge of optimizing both control accuracy and response speed in trajectory tracking for lower-limb exoskeleton hip robots. We introduce an optimization strategy that integrates the Sparrow Search Algorithm (SSA) with fuzzy Proportional-Integral-Derivative (PID) control. This approach addresses the inefficiencies and time-consuming process of manual parameter tuning, thereby improving trajectory tracking performance. First, recognizing the complexity of hip joint motion, which involves multiple degrees of freedom and intricate dynamics, we employed the Lagrangian method. This method is particularly effective for handling nonlinear systems and simplifying the modeling process, allowing for the development of a dynamic model for the hip joint. The SSA is subsequently utilized for the online self-tuning optimization of both the proportional and quantization factors within the fuzzy PID controller. Simulation experiments confirm the efficacy of this strategy in tracking hip joint trajectories during flat walking and standing hip flexion rehabilitation exercises. Experimental results from diverse test populations demonstrate that SSA-fuzzy PID control improves response times by 27.8% (for flat walking) and 30% (for standing hip flexion) when compared to traditional PID control, and by 6% and 2%, respectively, relative to fuzzy PID control. Regarding tracking accuracy, the SSA-fuzzy PID approach increases accuracy by 81.4% (for flat walking) and 80% (for standing hip flexion) when compared to PID control, and by 57.5% and 56.8% relative to fuzzy PID control. The proposed strategy significantly improves both control accuracy and response speed, offering substantial theoretical support for rehabilitation training in individuals with lower-limb impairments. Moreover, in comparison to existing methods, this approach uniquely tackles the challenges of parameter tuning and optimization, presenting a more efficient solution for trajectory tracking in exoskeleton systems.

## 1. Introduction

The rapid advancements in rehabilitation medicine and intelligent robotics have led to significant interest in lower-limb exoskeleton robots [1,2,3], particularly for their wide-ranging applications in the rehabilitation of patients with lower-limb motor dysfunction. Among these, ensuring body balance and achieving smooth gait walking are critical, with the precision of trajectory tracking control of the hip joint—an essential hub of lower-limb movement—playing a pivotal role. However, the complexity of human movement, variations in individual physiological characteristics, and the impact of external disturbances pose significant challenges for trajectory tracking control in lower-limb exoskeleton hip robots [4,5,6]. Consequently, investigating the achievement of high-precision trajectory tracking control for the lower-limb exoskeleton hip joint is of paramount importance for the rehabilitation of patients with lower-limb motor dysfunction.

Commonly employed methods for trajectory tracking control in lower-limb exoskeleton robots include model predictive control [7], PID control [8], sliding mode control [9,10], fuzzy control [11], and iterative learning control [11,12]. Among these, PID control is a simple and effective method widely used in industrial applications; however, it faces challenges in achieving precise control in complex systems. To address this, some researchers have applied intelligent algorithms to optimize PID parameters. For example, literature [13] employed artificial neural networks as an optimization tool to adjust the gains of the PID controller for multi-joint lower-limb exoskeletons in gait rehabilitation, resulting in improved tracking accuracy. However, overshooting remains a concern. Literature [14] introduced an enhanced single neural network PID controller to improve the stability of leg joint control in footed robots; however, its effectiveness across various motion scenarios was not tested. Literature [15] utilized a differential evolution algorithm to optimize PID parameters for rapid tracking of a given trajectory. While effective for simple paths, it exhibits limitations in tracking complex paths. Literature [16] proposed a hybrid PID and slip film control algorithm, which effectively achieves trajectory tracking of the exoskeleton mechanical leg and satisfies specific rehabilitation needs. However, no tracking tests have been conducted on the actual movement trajectories of the human body, presenting a limitation. Although significant improvements have been made to PID control, there remain notable shortcomings in trajectory tracking control for lower-limb exoskeleton robots. In recent years, fuzzy control has been increasingly applied in lower-limb trajectory tracking due to its robustness and ability to manage systems with low model accuracy. Sun [17] and colleagues proposed a simplified adaptive fuzzy system to mitigate chattering in the control process of lower-limb exoskeleton robots, demonstrating effective trajectory tracking performance through experimental validation. Wang [18] and colleagues developed a fuzzy PID controller for a lower-limb rehabilitation robot, using fuzzy rules to adjust the PID control parameters and enhance trajectory tracking performance. However, existing studies [19,20,21] indicate that the quantization and proportional factors in fuzzy PID controllers are crucial to the overall control system. Typically, these factors are determined through trial-and-error methods, which are inefficient and often fail to yield optimal trajectory control results. To further improve the reliability and safety of robot system modeling and control, recent studies have proposed research related to exoskeletons. For example, Krakhmalev et al. (2021) introduced a parallel computing algorithm for object-oriented modeling, which effectively supports the modeling of robotic systems and enhances the performance and stability of exoskeleton control systems [22]. Additionally, Tsapin et al. (2024) explored the application of machine learning methods in industrial robot system safety and provided new insights for the safety control of exoskeleton systems [23]. These studies provide important theoretical support for the modeling, control, and safety of exoskeleton robot systems and are worth further exploration in future work. In summary, while existing control methods have made progress in improving trajectory tracking performance for lower-limb exoskeleton robots, further advancements are needed to better balance control accuracy and response speed.

In response to these challenges and to improve trajectory tracking accuracy across various motion scenarios, this paper proposes a controller that integrates the Sparrow Search Algorithm (SSA) with fuzzy PID control. The proposed controller is designed to enhance both accuracy and response speed in lower-limb exoskeleton hip robots during common rehabilitation actions, such as ground walking and standing hip flexion, while simultaneously reducing the complexity of controller parameterization. This approach leverages the advantages of the SSA, including its robust optimization capabilities, high solution efficiency, and strong performance, to improve system effectiveness. This paper presents a controller based on the Sparrow Search Algorithm (SSA) and fuzzy PID control, specifically designed to enhance trajectory tracking accuracy and response speed for two common rehabilitation actions: human flat-floor walking and standing hip flexion. The objective of this work is to enhance the overall performance of lower-limb exoskeleton hip robots, thereby facilitating more effective lower-limb rehabilitation.

## 2. Dynamic Analysis of a Lower-Limb Exoskeleton Hip Robot

**Figure 1:**
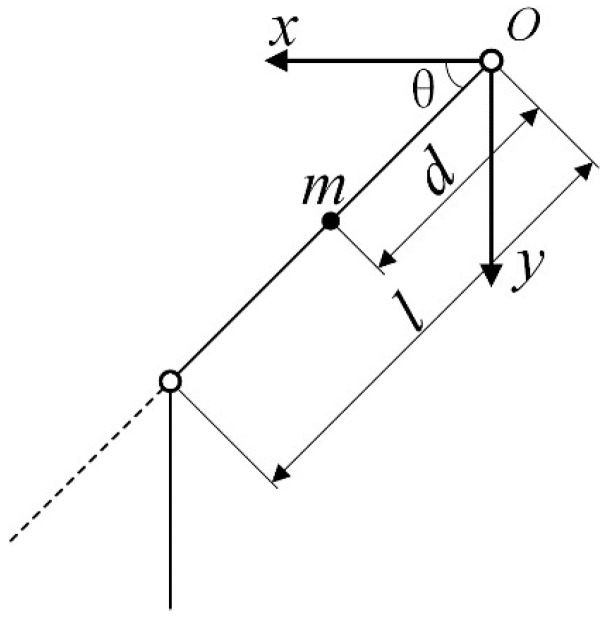
Dynamic model of the hip joint.

Existing research in human lower-limb biomechanics indicates that movement predominantly occurs within the sagittal plane. Consequently, this paper utilizes the Lagrangian method to analyze the dynamics of the lower-limb hip joint within the sagittal plane. A schematic representation of the model is provided in Figure 1.

Let l denote the length of the thigh segment of the lower-limb exoskeleton robot, m the mass, d the distance from the center of mass to the center of rotation, I the rotational inertia of the thigh segment about its center of mass, θ the angle of rotation at the hip joint, $\dot{\theta }$ the angular velocity, and $\ddot{\theta }$ the angular acceleration of the hip joint. The control torque applied to the hip joint is denoted by τ, and the gravitational acceleration is denoted by g.

Let the coordinates of the center of mass of the thigh segment be ($x_{1}$, $y_{1}$), then the expression for the position of the center of mass is:

(1) $$\begin{array}{l} x_{1}=dcos\theta \\ y_{1}=dsin\theta \end{array}$$

The square of the velocity of the thigh segment’s center of mass is given by:

(2) $$v_{c}^{2}={\dot{x}}_{1}^{2}+{\dot{y}}_{1}^{2}=d^{2}{\dot{\theta }}^{2}$$

The kinetic energy of the hip joint is given by:

(3) $$E_{k}=\frac{1}{2}mv_{c}^{2}+\frac{1}{2}I\omega^{2}=\frac{1}{2}md^{2}{\dot{\theta }}^{2}+\frac{1}{2}I{\dot{\theta }}^{2}$$

The potential energy of the hip joint is given by:

(4) $$E_{p}=mgdsin\theta$$

The Lagrangian function for the lower-limb exoskeleton hip robot is given by:

(5) $$L=E_{k}-E_{p}=\frac{1}{2}md^{2}{\dot{\theta }}^{2}+\frac{1}{2}I{\dot{\theta }}^{2}-mgdsin\theta$$

The Lagrangian kinetic equation for the lower-limb exoskeleton thigh linkage is:

(6) $$\tau =\frac{d}{dt}\frac{\partial L}{\partial \dot{\theta }}-\frac{\partial L}{\partial \theta }=md^{2}\ddot{\theta }+I\ddot{\theta }+mgdcos\theta$$

## 3. Control System Design

### 3.1. PID Controller

**Figure 2:**
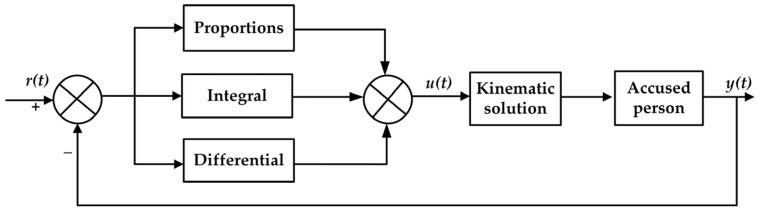
Structure of the PID control system.

The traditional PID controller is widely used in industrial applications because of its straightforward structure and proven control effectiveness. A diagram of the control structure is presented in Figure 2.

In the figure, r(t) represents the desired input value, y(t) is the actual output of the system, and u(t) denotes the output of the PID controller at time t. The control law is given by:

(7) $$u(t)=k_{p}e(t)+k_{i}\int_{0}^{t}e(t)dt+k_{d}\frac{de(t)}{dt}$$

Equation (7) is based on classical PID theory. Here, k_p_, k_i_, and k_d_ represent the proportional, integral, and differential coefficients of the PID controller, respectively, and e(t) denotes the error between the desired input value and the actual output, expressed as:

(8) e(t)=r(t)−y(t)

### 3.2. Fuzzy PID Controller Design

As this paper concentrates exclusively on modeling the hip joint within the sagittal plane, this limitation, along with external disturbances, reduces the accuracy of the model. As a result, achieving optimal control performance using traditional PID control becomes difficult. Furthermore, to ensure the safety of patients utilizing lower-limb exoskeleton devices during rehabilitation exercises and to prevent secondary injuries from improper control, the fuzzy PID controller proposed in this study incorporates fuzzy rules for adaptive adjustment. This approach eliminates the dependence on fixed parameter settings typical of traditional PID controllers. This design provides the fuzzy PID controller with greater flexibility, enabling it to rapidly adapt to dynamic changes within the controlled system.

**Figure 3:**
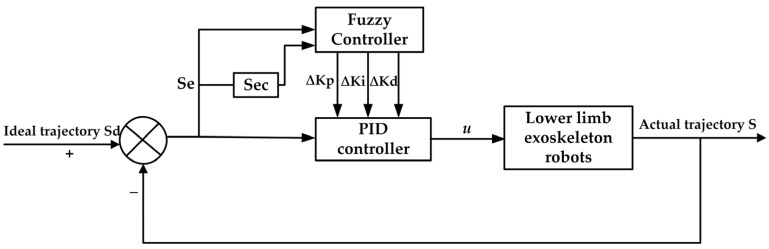
Principle of the fuzzy PID controller structure.

The fuzzy PID controller builds upon the traditional PID control by dynamically adjusting the proportional, integral, and differential coefficients through fuzzification, fuzzy reasoning, and defuzzification processes. This approach aims to enhance control performance. The structural schematic of the fuzzy PID controller is presented in Figure 3.

In the figure, S_d_ represents the ideal trajectory of the human lower-limb hip joint movement, while S denotes the actual trajectory. S_e_ is the deviation between the ideal and actual values of the hip joint movement trajectory angle, expressed as:

(9) $$s_{e}=s_{d}-s$$

S_ec_ represents the rate of change in the angular deviation in the hip joint movement trajectory, expressed as:

(10) $$s_{ec}=\frac{ds_{e}}{dt}$$

Δk_p_, Δk_i_, and Δk_d_ represent the correction parameters applied to the PID controller, derived through fuzzy reasoning. At this point, each parameter of the PID controller is expressed as:

(11) $$\begin{array}{l} k_{p}=k_{p1}+\Delta k_{p}\\ k_{i}=k_{i1}+\Delta k_{i}\\ k_{d}=k_{d1}+\Delta k_{d} \end{array}$$

Here, k_p1_, k_i1_, and k_d1_ represent the original parameters of each component of the PID controller, respectively. u is the output value resulting from the combined effect of fuzzy control and PID control, and is expressed as:

(12) $$u(t)=(k_{p1}+\Delta k_{p})e(t)+(k_{i1}+\Delta k_{i})\int_{0}^{t}e(t)dt+(k_{d1}+\Delta k_{d})\frac{de(t)}{dt}$$

Equation (12) is an extension of the PID controller incorporating fuzzy control, designed and innovated based on the specific requirements of the lower-limb exoskeleton system. This approach provides adaptive adjustment capabilities for addressing the control challenges of the lower-limb exoskeleton.

**Figure 4:**
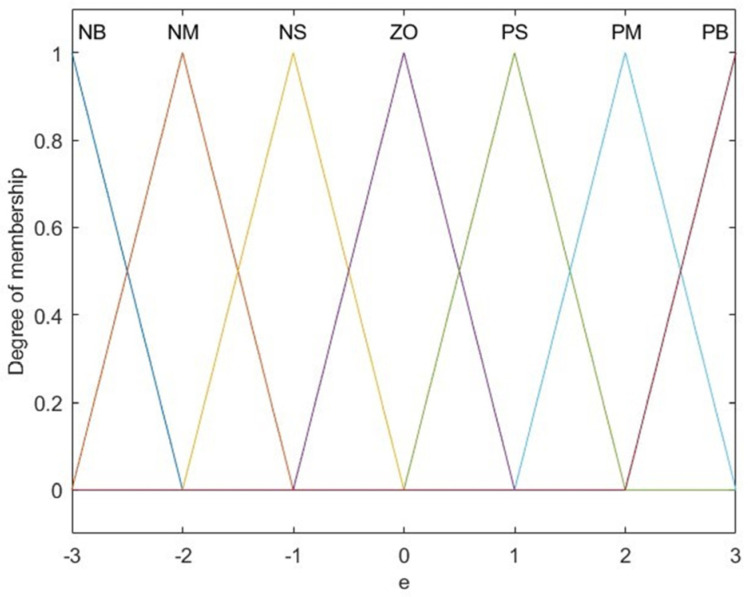
Membership function of input and output variables.

The fuzzy PID controller proposed in this paper uses the hip joint rotation angle deviation Se and the rate of change in deviation Sec as inputs, while the outputs are Δk_p_, Δk_i_, and Δk_d_. The fuzzy sets for the input and output variables are represented by {NB (Negative Large), NM (Negative Medium), NS (Negative Small), ZE (Zero), PS (Positive Small), PM (Positive Medium), and PB (Positive Large)}, with the input-output variables’ domain ranging from −3 to 3. The quantization factors for the input variables S_e_ and S_ec_ in the fuzzy PID controller are k_e_ and k_ec_, respectively. The scaling factors for the output variables Δk_p_, Δk_i_, and Δk_d_ are denoted as Ck_p_, Ck_i_, and Ck_d_, respectively. Given that the triangular membership function offers both simplicity and high efficiency, it is employed as the dependency function for the input and output variables, as illustrated in Figure 4.

The detailed fuzzy rules governing the relationships between the parameters are provided in Table 1.

**Figure 5:**
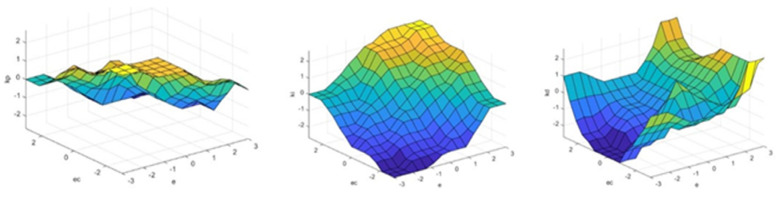
Fuzzy surfaces of Δk_p_, Δk_i_, and Δk_d_.

The variation surface of the output variables for the fuzzy PID controller is shown in Figure 5.

### 3.3. SSA-Fuzzy PID Control System Design

Due to the inherent nonlinearity, multimodality, and high-dimensionality of the control problems in exoskeleton systems, traditional optimization algorithms often struggle to achieve optimal results in complex motion trajectory tracking tasks. To address these challenges, this study adopts the Sparrow Search Algorithm (SSA) as an optimization tool. Leveraging its global search capabilities, SSA effectively identifies superior solutions within complex optimization spaces, while also being able to handle parameter fluctuations and external disturbances. This significantly enhances the adaptability and stability of the control system in dynamic environments.

#### 3.3.1. The Sparrow Search Algorithm (SSA)

The Sparrow Search Algorithm (SSA) is a novel swarm intelligence optimization technique that classifies the sparrow population into discoverers, joiners, and scouts, based on their foraging and anti-predator behaviors. The transfer of information and movement of positions among discoverers, joiners, and scouts facilitate the efficient exploration of the global optimal solution.

Let n represent the number of sparrows; thus, the population can be expressed as:

(13) $$X={\left[x_{1},x_{2},\cdots ,x_{n}\right]}^{T},x_{i}=\left[x_{i,1},x_{i,2},\cdots ,x_{i,d}\right]$$

Here, n and d represent the number of sparrows and the dimension of the optimization variable, respectively.

The fitness values for all sparrows are given by the following expression:

(14) $$\left\{\begin{array}{c} F_{X}={\left[f\left(x_{1}\right),f\left(x_{2}\right),\cdots ,f\left(x_{n}\right)\right]}^{T}\\ f\left(x_{i}\right)=\left[f\left(x_{i,1}\right),f\left(x_{i,2}\right),\cdots ,f\left(x_{i,d}\right)\right] \end{array}\right.$$

Here, f represents the fitness value. A subset of sparrows with the optimal fitness values is designated as discoverers, and their positions are updated as follows:

(15) $$X_{i,j}^{t+1}=\left\{\begin{array}{ll} X_{i,j}^{t}\cdot exp\left(-\frac{i}{\alpha \cdot iter_{max}}\right) & \;if\;R_{2}<ST\\ X_{i,j}^{t}+Q\cdot L & \;if{\;R}_{2}\geq ST \end{array}\right.$$

where t and itermax represent the current iteration number and the maximum iteration number, respectively; X_i,j_ represents the position of the i-th sparrow in the j-th dimension; α is a random number in the range (0, 1); $R_{2}$ is the warning value, taking values in the range [0, 1]; ST is the safety value, ranging from [0.5, 1]; Q is a random number following a normal distribution in the range [0, 1]; L is a 1 × d dimensional matrix with each element being a random number. When $R_{2}$ < ST, it indicates that no predators are present in the foraging environment, allowing the discoverer to conduct extensive search operations. Conversely, if $R_{2}$ ≥ ST, it signifies that some sparrows in the population have detected a predator and alerted the others. In this case, all sparrows must rapidly fly to safer locations for foraging.

In sparrow populations, the roles of joiners and discoverers can interchange, while maintaining a constant proportion of each within the population, thereby enhancing food searching efficiency. The position of the joiner is updated as follows:

(16) $$X_{i,j}^{t+1}=\left\{\begin{array}{ll} Q\cdot exp\left(\frac{X_{worst}-X_{i,j}^{t}}{i^{2}}\right) & if\;i>\frac{n}{2}\\ X_{p}^{t+1}+\left|X_{i,j}^{t}-X_{p}^{t+1}\right|\cdot A^{+}\cdot L & otherwise \end{array}\right.$$

where X_p_ represents the optimal position currently occupied by the discoverer, and X_worst_ denotes the global worst position at that moment. A is a 1 × d matrix, where each element is randomly assigned a value of 1 or −1, and $A^{+}=A^{T}{\left(AA^{T}\right)}^{-1}$. When i > n/2, it indicates that the i-th joiner, with a lower fitness value, is not obtaining food and is in a highly hungry state. Consequently, it needs to fly to another location to forage and gather more energy.

Approximately 10–20% of the sparrows in the population are responsible for scouting and detecting hazards in their environment. The positions of the scouts are updated as follows:

(17) $$X_{i,j}^{t+1}=\left\{\begin{array}{ll} X_{best}^{t}+\beta \cdot \left|X_{i,j}^{t}-X_{best}^{t}\right| & if\;f_{i}>f_{g}\\ X_{i,j}^{t}+K\cdot \left(\frac{\left|X_{i,j}^{t}-X_{worst}^{t}\right|}{\left(f_{i}-f_{w}\right)+\varepsilon }\right) & if\;f_{i}=f_{g} \end{array}\right.$$

where $X_{best}$ represents the current global optimal position. β, a step control parameter, is a random number drawn from a normal distribution with a mean of 0 and a variance of 1. K ∈ [−1, 1] is a random number, and $f_{i}$ denotes the current fitness value of an individual sparrow. $f_{g}$ and $f_{w}$ represent the current global optimal and worst fitness values, respectively. ε is a small constant introduced to avoid division by zero in the denominator.

#### 3.3.2. Process for Constructing the SSA-Fuzzy PID Control System

**Figure 6:**
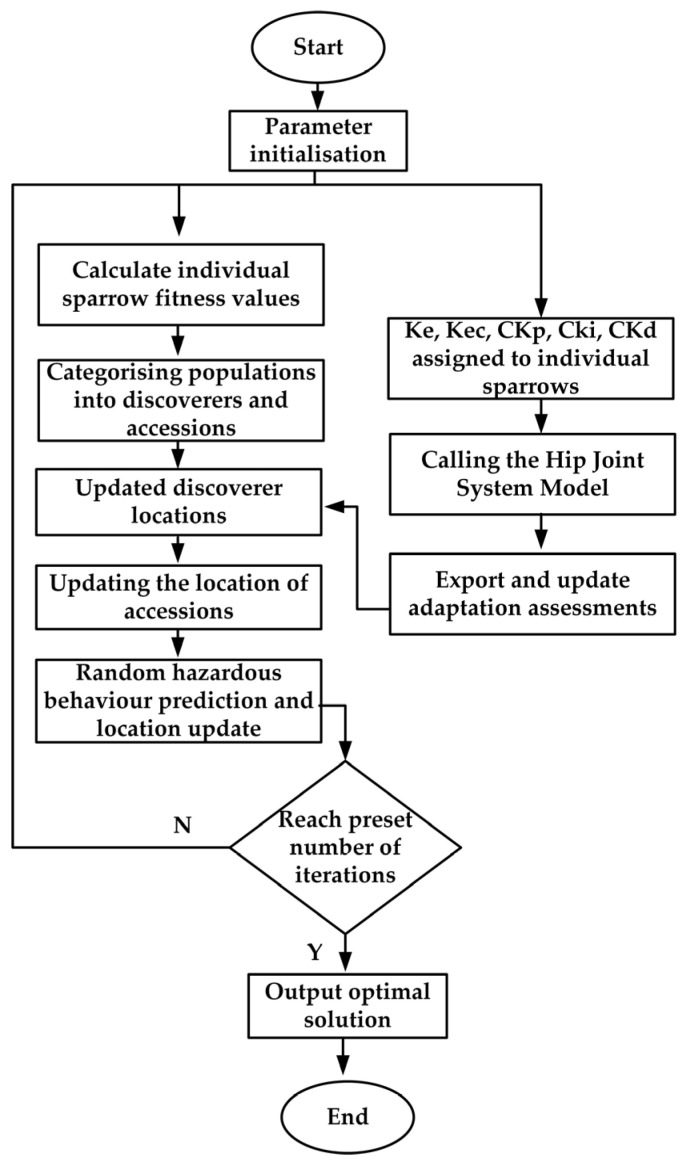
Flowchart of SSA-fuzzy PID parameter optimization.

Due to the SSA algorithm’s strong global search capability and rapid optimization speed, it is employed for the online iterative optimization of the quantification factor in the input variables and the proportional factor in the output variables of the fuzzy PID control. This approach addresses the inefficiency and time-consuming nature of manual tuning, which traditionally depends on the expertise of specialists over extended periods. The flowchart illustrating the application of the SSA algorithm to optimize these two factors is shown in Figure 6.

In this study, we first construct the fuzzy PID control system model as outlined earlier, and subsequently apply the SSA algorithm for online iterative optimization. The specific optimization steps are outlined below:

(1) Set the initial sparrow population size to 50, the number of iterations to 50, the safety value ST to 0.8, the discoverer proportion PD to 0.2, the alert proportion SD to 0.2, and the sampling period to 0.01 s.
(2) Establish the performance index evaluation criterion as the fitness function. The adaptation function is defined as the absolute value of the product of time t and the angular deviation of the hip joint movement trajectory S_e_(t), expressed as follows:

(18) $$J=\int_{0}^{t}t\left|s_{e}(t)\right|dt$$

(3) The SSA algorithm is implemented in MATLAB R2020a for joint simulation with the developed fuzzy PID hip control system, which outputs the optimized parameters. The corresponding code is as follows:
  y_fitness(1,iter) = fMin;
  K_p(1,iter) =
bestX(1);
  K_i(1,iter) =
bestX(2);
  K_d(1,iter) =
bestX(3);
  K_e(1,iter) =
bestX(4);
  K_ec(1,iter) =
bestX(5);

(4) Throughout the iteration process, the position and fitness function of each sparrow are continuously updated, and the corresponding optimized parameters are recorded. The performance indices are evaluated, where a smaller fitness value indicates a smaller angular deviation in the hip joint motion trajectory, reflecting improved control performance. Once the maximum number of iterations is reached, the algorithm terminates, and the resulting optimal parameters are applied to the fuzzy PID hip joint control system for verification.

## 4. Simulation Analysis and Results

### 4.1. Determination of Parameters for the SSA-Fuzzy PID Control System

In this study, the parameter optimization of the SSA-fuzzy PID control system is conducted in the MATLAB R2020a environment. Initially, the simulation model is configured with the relevant parameters, and the Simulink solver is set to a fixed step size of 0.01. Next, the parameters for the hip joint motion model are defined: the thigh mass m is set to 6 kg, the thigh linkage length l for the lower-limb hip joint is set to 0.46 m, in accordance with the dimensions specified in GB/T 10000-2023 Chinese Adult Human Body Dimensions [24]. The moment of inertia of the thigh linkage mass I is set to 0.3174 kg∙m^2^, and the gravitational acceleration g is assumed to be 10 m/s^2^. Given that the trajectory of the human lower-limb hip joint resembles a sinusoidal curve, the simulation test in this study uses a sinusoidal signal as the input, tracking the target curve to validate the effectiveness of the SSA-fuzzy PID control strategy.

**Figure 7:**
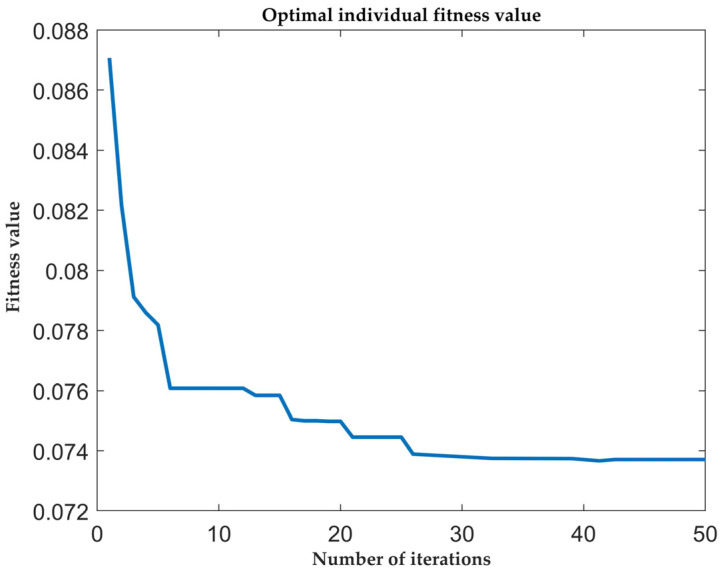
Iteration plot of SSA algorithm adaptation degree.

**Figure 8:**
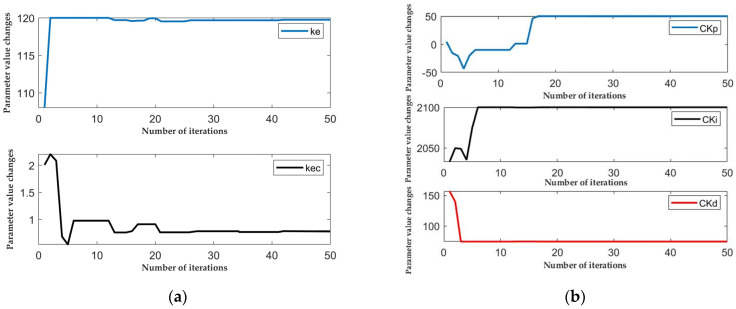
Parameter variations in the optimized SSA algorithm system. (**a**) Change in Ke and Kec parameters; (**b**) change in Ckp, Cki and Ckd parameters.

Based on the previous Equations (6), (7), (12) and (18), and all the equations in Section 3.3.1, we draw the following conclusion from the SSA-fuzzy PID parameter optimization simulation results: the SSA algorithm reaches the minimum fitness value and finds the optimal solution at the 26th iteration. The SSA algorithm fitness iteration graph and the changes in system parameters optimized by the SSA algorithm are shown in Figure 7 and Figure 8.

Table 2 presents the comparison of parameter values between the fuzzy PID controller and the SSA-fuzzy PID controller. It is evident that the parameters of the optimized SSA-fuzzy PID controller exhibit significant differences when compared to those of the conventional fuzzy PID controller. Specifically, the values of ke and Cki in the SSA-fuzzy PID controller show a notable increase, while kec decreases substantially, suggesting that the optimized parameters are better aligned within the ideal value range identified during the global search. This optimization process eliminates the randomness and inefficiency associated with traditional fuzzy PID control, which relies on manual parameter tuning, and instead achieves a more rational parameter configuration through an online iterative optimization method.

### 4.2. Comparative Analysis of Simulation Results for Three Control Systems

**Figure 9:**
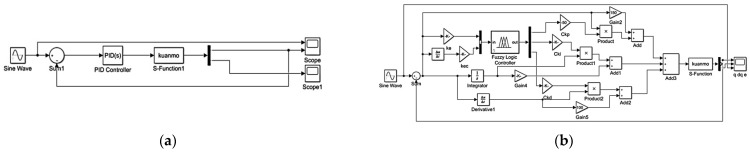
Simulation models. (**a**) Proportional-Integral-Derivative (PID) controller; (**b**) fuzzy PID controller.

**Figure 10:**
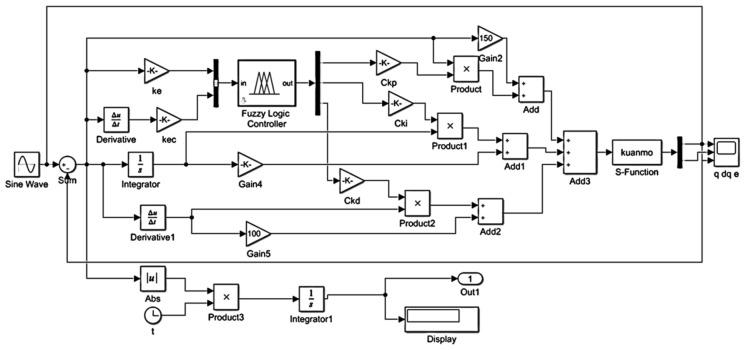
Simulation model of the Sparrow Search Algorithm (SSA)-based fuzzy PID controller.

To assess the performance of the proposed SSA-fuzzy PID controller in trajectory tracking control for the lower-limb exoskeleton hip robot, a series of simulation tests were conducted. These tests compared the PID controller, fuzzy PID controller, and SSA-fuzzy PID controller on two distinct trajectories: the hip joint movement during human flat walking and the hip flexion movement during standing. The simulation models for the PID controller, fuzzy PID controller, and SSA-fuzzy PID controller are illustrated in Figure 9 and Figure 10.

#### 4.2.1. Trajectory Tracking Test of Hip Joint Motion During Human-Level Walking Using Three Control Methods

**Figure 11:**
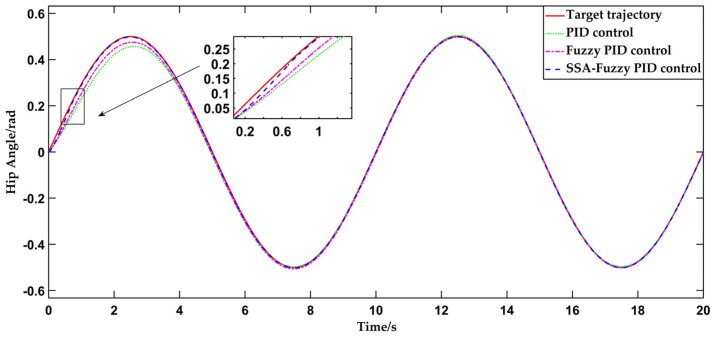
Comparison of hip joint motion trajectory tracking curves during human-level walking using three control methods.

This test uses the sinusoidal curve, as described in Section 4.1, as the input for trajectory tracking, implemented through the PID controller, fuzzy PID controller, and SSA-fuzzy PID controller. The simulation duration is set to 20 s, with the resulting data presented in Figure 11.

**Figure 12:**
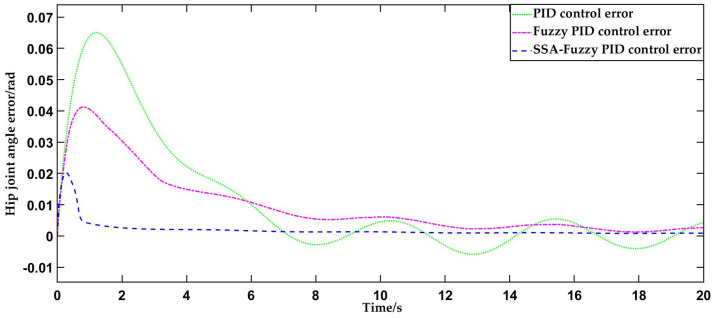
Comparison of tracking errors in hip joint motion trajectory during human-level walking using three control methods.

As depicted in Figure 11 and Figure 12, the traditional PID control begins to align with the desired hip joint motion trajectory at 3.2 s, successfully tracking the motion trajectory during human-level walking on flat ground. The steady-state error in hip joint motion is approximately 0.005854 rad. The fuzzy PID controller aligns with the desired hip joint motion trajectory in 2 s, improving the response time by 1.2 s compared to traditional PID control. The error in hip joint angle stabilizes at approximately 0.004904 rad once the system reaches equilibrium. The SSA-fuzzy PID controller aligns with the desired hip joint motion trajectory in just 0.7 s, with the hip joint angle error stabilizing at a constant value of 0.001840 rad once the system reaches stability. In comparison to both PID control and fuzzy PID control, the SSA-fuzzy PID controller demonstrates superior trajectory tracking accuracy and faster response times when tracking the hip joint trajectory during human-level walking on flat ground.

#### 4.2.2. Trajectory Tracking Test of Standing Hip Flexion Motion Using Three Control Methods

**Figure 13:**
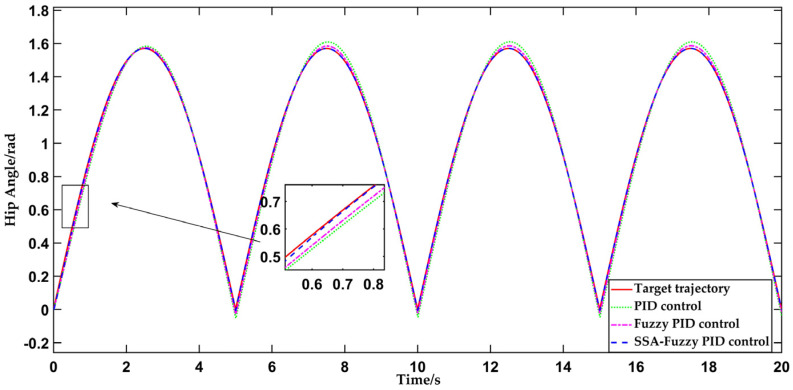
Comparison of trajectory tracking curves for standing hip flexion motion using three control methods.

Given that the standing hip flexion trajectory approximates the positive half-cycle of a sinusoidal curve, the positive half-cycle is selected as the desired trajectory for the standing hip flexion trajectory tracking test. The target trajectory of the hip joint is then tracked using PID control, fuzzy PID control, and SSA-fuzzy PID control, respectively. The simulation duration is 20 s, with the simulation process depicted in Figure 13.

**Figure 14:**
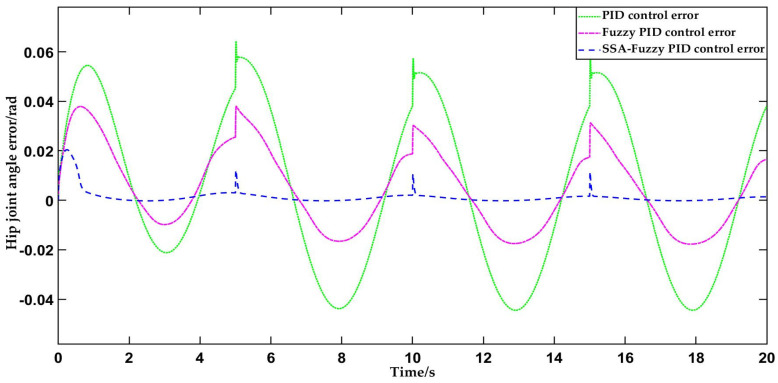
Comparison of tracking errors in standing hip flexion trajectory using three control methods.

As shown in Figure 13 and Figure 14, the traditional PID control begins to align with the desired hip joint motion trajectory at 2 s, successfully tracking the standing hip flexion trajectory. The steady-state error in hip joint motion remains within the range of −0.04369 to 0.06 rad. The fuzzy PID controller aligns with the desired hip joint motion trajectory at 1.8 s, and the steady-state error in hip joint motion remains within the range of −0.01654 to 0.03724 rad. The SSA-fuzzy PID controller tracks the standing hip flexion motion trajectory at 0.65 s, with the steady-state error in hip joint motion stabilized within the range of −0.0001914 to 0.01175 rad. Compared to both PID control and fuzzy PID control, the SSA-fuzzy PID controller demonstrates higher trajectory tracking accuracy and faster response times in tracking the standing hip flexion trajectory.

## 5. Lower-Limb Exoskeleton Hip Robot Trajectory Tracking: Empirical Testing

### 5.1. Construction of the Experimental Platform

**Figure 15:**
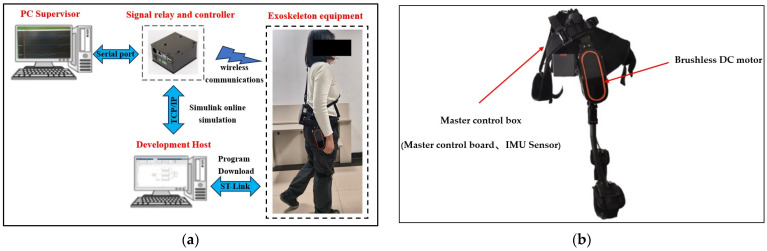
Experimental platform for the lower-limb exoskeleton hip robot. (**a**) Experimental platform; (**b**) lower-limb exoskeleton hip robot.

To evaluate the control performance of the SSA-fuzzy PID controller for trajectory tracking in a lower-limb exoskeleton hip robot, an experimental platform was constructed, as shown in Figure 15. The platform includes a development host, a PC upper computer, a signal relay and control box, an IMU sensor, a main controller, and a brushless DC motor.

Based on the GB/T 10000-2023 “Body Dimensions of Chinese Adults” statistics, adult males with heights between 177 cm and 180 cm correspond to the 90th and 95th percentiles, respectively, while males weighing 80 kg fall within the 90th percentile. For adult females, a height of 165 cm places them in the 90th percentile, while a weight of 55 kg corresponds to the 50th percentile, representing the average weight for women. To more comprehensively represent the height and weight distribution of adult males and females, and to ensure the applicability and generalizability of the experimental results, this study selected three healthy test subjects (two males and one female) with heights ranging from 165 to 183 cm and weights between 55 and 80 kg. Subject A, a female, had a height of 165 cm and a weight of 55 kg; Subject B, a male, had a height of 175 cm and a weight of 75 kg; and Subject C, a male, had a height of 183 cm and a weight of 80 kg.

Prior to the experiment, the code generated in Simulink for PID control, fuzzy PID control, and SSA-fuzzy PID control was sequentially downloaded to the lower-limb exoskeleton hip robot using the ST-LINK downloader. A wireless module on the exoskeleton was paired with a PC host, allowing for the recording and observation of the hip joint movement angles using a serial port tool.

**Figure 16:**
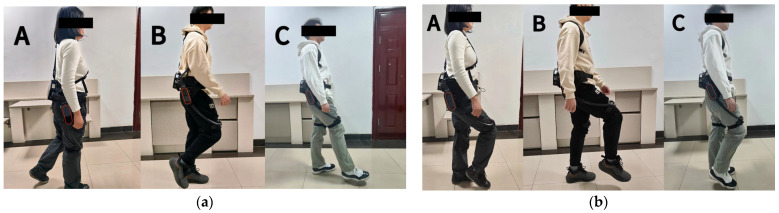
Motion data acquisition process for Testers A, B, and C wearing the lower-limb exoskeleton device. (**a**) Acquisition of motion data for Testers A, B, and C while walking on level ground; (**b**) collection of standing hip flexion movement data for Testers A, B, and C.

The data acquisition process involved three testers wearing the lower-limb exoskeleton hip device, walking and performing standing hip flexion movements on flat ground for 20 s. Motion data were randomly collected through posture sensors placed on the testers’ hip joints during these activities. The collected data were processed using Kalman filtering and MATLAB R2020a fitting to evaluate the lower-limb exoskeleton’s trajectory tracking performance in various motion scenarios. Figure 16 illustrates the motion data acquisition process for the three testers wearing the lower-limb exoskeleton device.

### 5.2. Analysis of Measured Results

#### 5.2.1. Hip Joint Trajectory Tracking Test for Testers A, B, and C During Level Walking Under Three Control Methods

**Figure 17:**
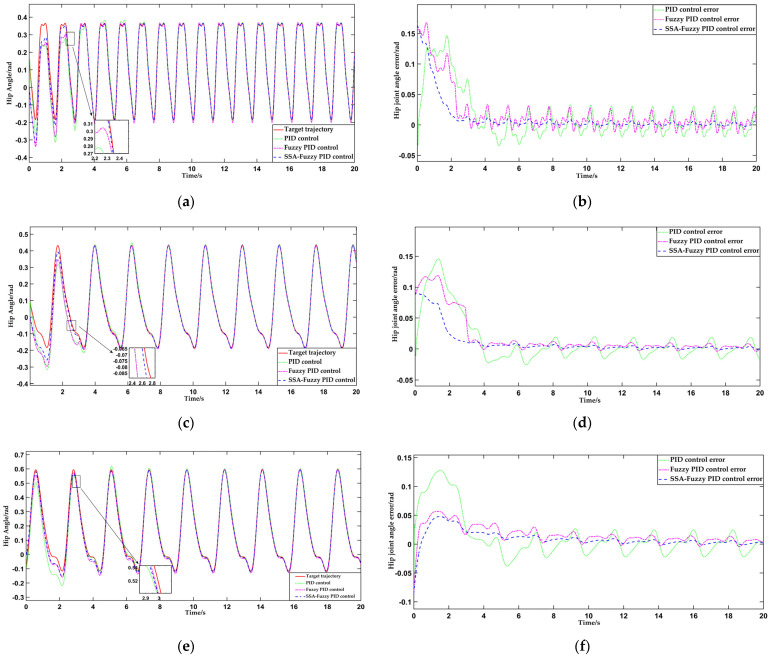
Comparison of hip joint trajectories and tracking errors during level walking for Testers A, B, and C under the three control methods. (**a**) Comparison of hip joint trajectory tracking curves during level walking for Tester A; (**b**) comparison of hip joint trajectory tracking errors during level walking for Tester A; (**c**) comparison of hip joint trajectory tracking curves during level walking for Tester B; (**d**) comparison of hip joint trajectory tracking errors during level walking for Tester B; (**e**) comparison of hip joint trajectory tracking curves during level walking for Tester C; (**f**) comparison of hip joint trajectory tracking errors during level walking for Tester C.

As shown in Figure 17, which compares the hip joint motion trajectories and tracking errors for Testers A, B, and C during flat walking under the three control methods, the performance differences between the control methods are immediately apparent. For a more detailed quantitative analysis, Table 3 and Table 4 summarize the hip motion tracking errors and response times of the three control systems in the flat ground walking scenario.

Based on the data presented in Table 3 and Table 4, it can be concluded that, under identical initial error conditions during level walking, the PID control system demonstrates a longer response time due to the absence of optimized parameter settings. In contrast, the SSA-fuzzy PID control maintains faster response times and effectively tracks the hip joint motion trajectory during level walking across different populations. On average, the response time is 27.8% faster than PID control and 6% faster than fuzzy PID control. In terms of accuracy, the SSA-fuzzy PID control demonstrates a narrower steady-state error range compared to both PID and fuzzy PID control. The trajectory tracking accuracy is improved by 81.4% and 57.5%, respectively, when compared to PID and fuzzy PID control.

#### 5.2.2. Trajectory Tracking Test for Testers A, B, and C: Standing Hip Flexion Movement Trajectories Under Three Control Methods

**Figure 18:**
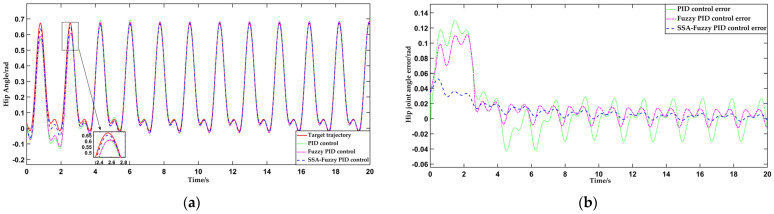

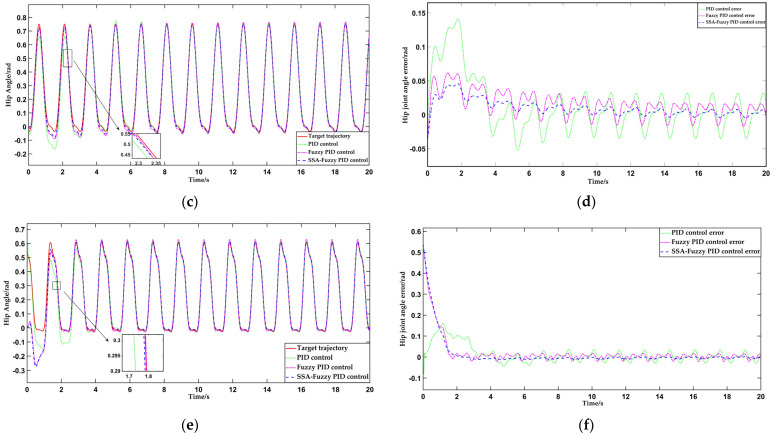
Comparison of the trajectories and tracking errors of standing hip flexion movements for Testers A, B, and C under the three control methods. (**a**) Comparison of trajectory tracking curves for Tester A’s standing hip flexion movement; (**b**) comparison of tracking errors for Tester A’s standing hip flexion trajectory; (**c**) comparison of trajectory tracking curves for Tester B’s standing hip flexion movement; (**d**) comparison of tracking errors for Tester B’s standing hip flexion trajectory; (**e**) comparison of trajectory tracking curves for Tester C’s standing hip flexion movement; (**f**) comparison of tracking errors for Tester C’s standing hip flexion trajectory.

Analyzing the data presented in Table 5 and Table 6, it is evident that the SSA-fuzzy PID control consistently achieves tracking of standing hip flexion movement trajectories for different individuals, with faster response times compared to the other two control systems, under the same initial error condition. The response time was, on average, 30% faster compared to PID control and 2% faster compared to fuzzy PID control. Regarding accuracy, the SSA-fuzzy PID control demonstrates a narrower steady-state error range compared to both PID and fuzzy PID control. The trajectory tracking accuracy is, on average, 80% and 56.8% higher compared to PID control and fuzzy PID control, respectively. See Figure 18.

### 5.3. Summary of Test Results for Two Different Sports Scenarios

The results presented in Section 5.2.1 and Section 5.2.2 demonstrate that the SSA-fuzzy PID control method outperforms other control strategies in trajectory tracking, achieving notable improvements in both level walking and standing hip flexion motion scenarios. Specifically, SSA-fuzzy PID control enhances response speed by reducing the average response time by 27.8% for level walking and 30% for standing hip flexion, compared to PID control. Additionally, compared to fuzzy PID control, the response time is improved by 6% for level walking and 2% for standing hip flexion, indicating superior dynamic response characteristics of SSA-fuzzy PID control.

Regarding tracking accuracy, SSA-fuzzy PID control significantly reduces steady-state errors. The accuracy of trajectory tracking is improved by 81.4% for level walking and 80% for standing hip flexion, compared to PID control, and by 57.5% for level walking and 56.8% for standing hip flexion, compared to fuzzy PID control. These results confirm that SSA-fuzzy PID control offers both faster response times and higher accuracy in trajectory tracking across different exercise scenarios, ultimately contributing to better rehabilitation outcomes for individuals undergoing lower-limb rehabilitation.

Although the SSA-fuzzy PID control method has demonstrated good performance in this study, it still has certain limitations in practical applications, primarily related to computational complexity, the convergence speed of SSA, and the complexity of fuzzy control rule design. These issues may affect the performance of the control system in complex motion scenarios.

## 6. Conclusions

This paper addresses the challenge of balancing control accuracy and response speed in lower-limb exoskeleton hip robots for trajectory tracking. A novel controller based on SSA-fuzzy PID control is proposed to optimize trajectory tracking accuracy and enhance overall control performance. The use of the sparrow search algorithm for online iterative parameter optimization of fuzzy PID control effectively addresses the inefficiency and time-consuming nature of manual parameter tuning. Simulation and trajectory tracking experiments conducted with different test groups confirm the rapid responsiveness and high accuracy of SSA-fuzzy PID control in tracking trajectories across various sports scenarios. This provides valuable theoretical support for trajectory tracking control in lower-limb exoskeletons and contributes to improving the rehabilitation outcomes for individuals engaging in lower-limb exercises.

In addition, SSA-fuzzy PID control not only has a significant impact on lower-limb rehabilitation but also holds promise for a wider range of assistive robotics and rehabilitation technologies. For instance, in fields such as smart prosthetics, elderly care robotics, and neurological rehabilitation systems, SSA-fuzzy PID control can substantially enhance the performance and reliability of these devices by providing more efficient real-time optimization and adaptive adjustments. This, in turn, contributes to improved patient outcomes and quality of life.

However, in the control of lower-limb exoskeletons, achieving high-precision control for the current two movement scenarios (flat walking and standing hip flexion) is not sufficient for a comprehensive evaluation of the algorithm’s effectiveness. To more objectively assess the performance of the SSA-fuzzy PID control algorithm, future research will extend to more complex movement scenarios, such as walking on stairs and uneven surfaces. These scenarios will be tested for trajectory tracking control, which will help further improve the algorithm’s adaptability and stability across different movement patterns, thereby enhancing the overall motion control performance of the exoskeleton and ultimately achieving more effective rehabilitation outcomes.

## Figures and Tables

**Table 1:** Fuzzy rule table for Δk_p_, Δk_i_, and Δk_d_.

Se/S_ec_	NB	NM	NS	ZO	PS	PM	PB
**NB**	PB/NB/PS	PB/NB/NS	PM/NM/NB	PM/NM/NB	PS/NS/NB	ZO/ZO/NM	ZO/ZO/PS
**NM**	PB/NB/PS	PB/NB/NS	PM/NM/NB	PS/NS/NB	PS/NS/NB	ZO/ZO/NM	ZO/ZO/PS
**NS**	PM/NB/ZO	PM/NM/NS	PM/NS/NS	PM/NS/NM	ZO/ZO/NS	NS/ZO/NS	NS/PS/ZO
**ZO**	PM/NM/ZO	PM/NM/NS	PM/NS/PM	ZO/ZO/NS	PS/PS/NS	NM/PM/NS	NM/PM/ZO
**PS**	PS/NM/ZO	PS/NS/ZO	ZO/ZO/ZO	NS/PS/ZO	NS/PS/ZO	NM/PM/ZO	NM/PB/ZO
**PM**	PS/ZO/PB	ZO/ZO/NS	NS/PS/PS	NM/PS/PS	NM/PM/PS	NM/PB/PS	NB/PB/PB
**PB**	ZO/ZO/PB	ZO/ZO/PM	NM/PS/PM	NM/PM/PM	NM/PM/PS	NB/PB/PS	NB/PB/PB

**Table 2:** Parameters of the fuzzy PID controller and SSA-fuzzy PID controller.

Controller Parameters	k_e_	k_ec_	Ck_p_	Ck_i_	Ck_d_
Fuzzy PID control	54.6	20	−50	55.8	2
SSA—Fuzzy PID Control	119.7467	0.7801	50	2100	75.0746

**Table 3:** Tracking response times of the three control systems for hip motion during flat walking.

Tester	Three Control Initial Errors (rad)	PID Response Time (s)	Fuzzy PID Response Time (s)	SSA-Fuzzy PID Response Time (s)
A	0.1716	3.610	2.391	2.338
B	0.09001	3.905	3.195	2.659
C	−0.08579	3.503	2.940	2.927

**Table 4:** Tracking steady-state error ranges of the three control systems for hip motion during level walking.

Tester/Steady-State Error Range (rad)	PID	Fuzzy PID	SSA-Fuzzy PID
A	−0.03363~0.03182	−0.009989~0.03005	−0.0006363~0.009561
B	−0.02527~0.01948	−0.001565~0.01236	−0.00001675~0.006947
C	−0.03825~0.02670	−0.002989~0.02770	−0.0008746~0.01498

**Table 5:** Tracking response times of the three control systems for standing hip flexion exercise.

Tester	Three Control Initial Errors (rad)	PID Response Time (s)	Fuzzy PID Response Time (s)	SSA-Fuzzy PID Response Time (s)
A	0.03700	3.597	2.739	2.512
B	−0.03521	3.838	2.373	2.311
C	0.5387	3.311	1.779	1.739

**Table 6:** Tracking steady-state error ranges of the three control systems for standing hip flexion exercise.

Tester/Steady-State Error Range (rad)	PID	Fuzzy PID	SSA-Fuzzy PID
A	−0.04268~0.02872	−0.009082~0.01883	−0.003384~0.008158
B	−0.05174~0.03408	−0.01407~0.02236	−0.003237~0.01759
C	−0.04336~0.03617	−0.01878~0.01744	−0.009866~0.001340

## Data Availability

The original contributions presented in this study are included in this article; further inquiries can be directed to the corresponding author.
